# First Case of Deficiency of Adenosine Deaminase 2 (DADA2) in Oman With a Novel Mutation: A Case Report

**DOI:** 10.7759/cureus.107895

**Published:** 2026-04-28

**Authors:** Nasra M Al Ramadhani, Ruqaiya Al Jashmi, Safiya Al-Abrawi

**Affiliations:** 1 Pediatric Rheumatology, Royal Hospital, Muscat, OMN

**Keywords:** bi-cytopenia, dada2, immunodeficiency, novel mutation, oman, tumor necrosis factor (tnf), vasculitis

## Abstract

Deficiency of adenosine deaminase 2 (DADA2) is a rare autosomal recessive disorder that results from biallelic loss-of-function mutations in the ADA2 gene. It is characterized by a spectrum of clinical features, predominantly vasculitis, autoinflammation, dysregulated immune function, and hematologic abnormalities, making it a challenging condition to diagnose. This case report presents the first documented case of DADA2 in Oman, highlighting a novel mutation in the ADA2 gene. It provides insights into the diagnostic process, therapeutic strategies, systemic evaluations, and family-focused management, contributing to the growing understanding of this rare disorder.

This is a case of an 18-month-old male toddler who presented with persistent bi-cytopenia and a history of febrile seizures. Genetic testing revealed a novel homozygous pathogenic variant in the ADA2 gene. The patient was managed with infliximab infusions and regular follow-up.

This report enhances the understanding of DADA2. The identification of a novel mutation in this case further highlights the growing insight into the pathogenesis of DADA2 and its clinical implications. Effective management requires genetic testing, systemic evaluation, and targeted therapy. Genetic counseling and family screening are crucial for early intervention and disease prevention. Further research is needed to improve diagnostics, treatments, and patient outcomes.

## Introduction

Deficiency of adenosine deaminase 2 (DADA2) is a monogenic autosomal recessive disorder resulting from biallelic loss-of-function mutations in the ADA2 gene located on chromosome 22q11.1 [1,2]. Since its initial description in 2014, approximately 95 DADA2-associated mutations have been reported in the literature to date [1-3]. ADA2 enzyme deficiency results in the accumulation of extracellular adenosine and dysregulation of the immune system, with increased production of inflammatory cytokines such as tumor necrosis factor alpha (TNF-α) and defective macrophage polarization, affecting both innate and adaptive immunity [1,4]. This dysregulation leads to vascular inflammation, often manifesting as vasculitis [1,2,5].

The earliest reported cases of DADA2 described a clinical phenotype resembling polyarteritis nodosa, with immunodeficiency and early-onset stroke [2,5]. However, with the evolving understanding of pathogenesis and the disease phenotype, the recognized clinical manifestations have broadened significantly to include a spectrum of features - predominantly systemic vasculitis, early-onset stroke, immunodeficiency, and hematologic abnormalities such as pure red cell aplasia, cytopenia, and bone marrow failure [3,6]. These variable presentations make DADA2 a particularly challenging condition to diagnose.

DADA2 is mostly diagnosed in childhood, although age at presentation can vary widely, ranging from infancy to adulthood. Diagnosis is guided by the consensus statements developed by the DADA2 Foundation and expert clinicians and is established through genetic testing or by measuring ADA2 enzyme activity [1,3]. Screening for DADA2 should be considered in patients with unexplained systemic vasculitis, early-onset stroke, immunodeficiency, or hematologic abnormalities [3,6]. Once diagnosis is confirmed, comprehensive systemic evaluation involves a detailed clinical examination, laboratory tests, and additional assessments including electrocardiogram, echocardiogram, brain MRI, abdominal ultrasound, and pulmonary function test to evaluate cardiac, neurological, abdominal, and respiratory involvement [3]. Bone marrow biopsy may also be considered in some cases [6]. This thorough evaluation aids in identifying the extent of the disease, tailoring treatment, and monitoring progression as well as response to therapy.

To date, no randomized clinical trials have been published exploring DADA2 management. Therefore, treatment recommendations are based on case reports, case series, and expert opinions [1,3,6,7]. The use of tumor necrosis factor (TNF) inhibitors has been shown to significantly reduce systemic inflammation and the risk of ischemic and hemorrhagic strokes [5,8]. Glucocorticoids, disease-modifying antirheumatic drugs (DMARDs), and biologics targeting IL-1 and IL-6 have been used in managing patients with DADA2, though with limited long-term efficacy and relapsing or refractory cases [1,6]. Allogenic hematopoietic stem cell transplantation (HSCT) has been used for cases with bone marrow failure, severe immunodeficiency, or refractory vascular involvement, showing significant clinical improvement and high survival rates [6,8].

Given the autosomal recessive inheritance pattern of DADA2, genetic counseling and family screening are crucial [1,3,6]. Genetic testing is recommended for all siblings and symptomatic relatives [3,6]. Early identification of asymptomatic carriers or affected individuals enables timely intervention and can prevent severe complications [3]. The prognosis of DADA2 varies widely depending on the severity of manifestations and the timing of diagnosis and treatment initiation [1,6]. This case report presents the first documented case of DADA2 in Oman with a novel mutation, highlighting the clinical presentation, diagnostic journey, therapeutic interventions, comprehensive monitoring, and genetic counseling.

## Case presentation

The patient was an 18-month-old male toddler, born at term via spontaneous vaginal delivery with a birth weight of 3.4 kg. He had an initial neonatal course complicated with hydrops fetalis and respiratory distress requiring admission to the neonatal intensive care unit, intubation, and ventilatory support. He was noted to have anemia and received a packed red blood cell (PRBC) transfusion before being discharged on day 10 of life.

At five weeks of age, he presented to the emergency department at the Royal Hospital, Oman, with severe symptomatic anemia (hemoglobin 6.9 g/dL) and neutropenia (white blood cell count 4.7x 10⁹/L; absolute neutrophil count 0.8x 10⁹/L) with preserved platelet count. Serial follow-up demonstrated persistent bi-cytopenia, characterized by recurrent anemia requiring multiple PRBC transfusions and ongoing mild to moderate neutropenia. A detailed history and physical examination were performed at that time. There was no history of recurrent infections, prolonged fever, failure to thrive, mucocutaneous ulcers, joint swelling or redness, or any history of stroke or neurological deficits. However, during subsequent follow-up, parents reported episodes of brief febrile tonic-clonic seizures. The parents are consanguineous (first cousins) and have four other healthy children, with no family history of genetic, hematologic, rheumatologic conditions, or immunodeficiency disorders. Physical examination revealed eczematous skin rash in the perioral area and over the extensor surfaces of bilateral lower limbs, with no other skin rashes. He appeared pale, with no jaundice, petechiae, or skin bruising. There was no lymphadenopathy. At presentation, his weight, length, and head circumference were within normal limits for age. Vital signs were all within normal limits for age. The examination was negative for any heart murmurs, hepatosplenomegaly, or abdominal masses. Joint examination was normal, and he had no neurological deficits.

During the initial evaluation, he was thoroughly investigated for possible hematological and infectious etiologies of bi-cytopenia (Table 1). A detailed hematological assessment, including anemia and hemolytic workup, as well as evaluation for hemoglobinopathies and bleeding disorders, was conducted and found unremarkable apart from a picture of alpha thalassemia trait, which is an incidental finding that is usually asymptomatic and does not explain the patient's clinical picture. Bone marrow examination was not performed as initial investigations did not indicate bone marrow failure requiring invasive evaluation. Infectious workup, including congenital TORCH (toxoplasmosis, others (syphilis, hepatitis B), rubella, cytomegalovirus, herpes simplex) screening and viral studies, was negative.

**Table 1:** Initial laboratory investigations. Abbreviations: Hb, hemoglobin; MCV, mean corpuscular volume; MCHC, mean corpuscular hemoglobin concentration; WBC, white blood cells; PLT, platelets; ESR, erythrocyte sedimentation rate; IgG, immunoglobulin G; IgA, immunoglobulin A; IgM, immunoglobulin M; IgE, immunoglobulin E.

Test item	Value	Normal range
Hb	7.4 g/dL	10-14.1 g/dL
MCV	76 fL	76-96 fL
MCHC	33.5 g/dL	31-35 g/dL
WBC	5.5 10*9/L	6-20 10*9/L
Neutrophils	0.9 10*9/L	1-8.5 10*9/L
PLT	313 10*9/L	140-400 10*9/L
Haptoglobin	638 mg/L	30-3000 mg/L
Reticulocyte count	3.8%	0.2-2%
Lactate dehydrogenase serum	397 IU/L	180-430 IU/L
Total bilirubin	10 umol/L	5-21 umol/L
Ferritin	384 ug/L	22-322 ug/L
ESR	35 mm/h	2-25 mm/h
C-reactive protein	8 mg/L	< 10 mg/L
Lymphocytes subset analysis		
T cell (CD3+)	2.12 10*9/L	2.2-8.3 10*9/L
Helper T cells (CD4+)	1.11 10*9/L	1-3.6 10*9/L
Cytotoxic T cells (CD8+)	0.89 10*9/L	0.6-2.2 10*9/L
CD4:CD8 ratio	1.25 10*9/L	1-3 10*9/L
B cells (CD19+)	0.67 10*9/L	0.7-1.3 10*9/L
Immunoglobulin panel		
IgG	12.1 g/L	4-12 g/L
IgA	0.57 g/L	0.15-1.8 g/L
IgM	0.83 g/L	0.3-2 g/L
IgE	54 iU/mL	0.4-352 iU/mL

Given the persistent cytopenia and inconclusive investigations, the patient continued to be followed closely. At 18 months of age, further evaluation for an underlying genetic etiology was pursued. Whole exome sequencing (WES) was done and identified a homozygous, likely pathogenic variant in the ADA2 gene (c.1442+2T>A variant), which is a novel mutation that has never been reported in the literature (Table 2).

**Table 2:** Whole exome sequencing report of the patient showing a homozygous mutation involving the ADA2 gene. OMIM: Online Mendelian Inheritance in Man

Gene (transcript)	Variant	Zygosity	Disease (OMIM)	Inheritance	Classification
ADA2	c.1442+2T>A	homozygous	Vasculitis, auto-inflammation, immunodeficiency, and hematologic defects syndrome	Autosomal recessive	Likely pathogenic

Following diagnosis, parents were counseled extensively about the rarity of this condition and that their son is the first diagnosed case in Oman. They were also educated about DADA2 genetic inheritance, clinical presentations, systemic evaluation for all possible manifestations, and the importance of early treatment initiation to prevent undesirable complications. They were counseled about the necessity of screening all siblings for DADA2 and any symptomatic family members, if any. Parents were provided with helpful sources to read more about this condition. Meanwhile, a comprehensive systemic evaluation was conducted in a timely manner to assess for DADA2-associated clinical manifestations. Baseline ECG showed normal regular sinus rhythm. Ophthalmological examination was also normal. Abdominal ultrasound was unremarkable for hepatosplenomegaly or portal hypertension. Brain MRI was initially planned but deferred after parental refusal of general anesthesia. Following further counseling, the family subsequently agreed to proceed with the study, and an appointment was scheduled. Immunological evaluation, including lymphocyte subset analysis, immunoglobulin levels, and antibody titers, was within normal limits. Borderline lymphocyte subset values were reviewed by the immunology team and were considered to have no clinical significance in the current clinical context.

The patient was started on infliximab infusion at a dose of 6 mg/kg (total dose 60 mg intravenously) every four weeks and received a total of six doses. Subsequently, due to compliance concerns and transportation difficulties related to long travel distance, he was transitioned to weekly etanercept subcutaneous injections. He remained on regular follow-up in our clinical demonstrated clinical stability with improvement in hemoglobin and neutrophil counts. No further blood transfusion was required after initiation of treatment.

## Discussion

This case report highlights the diagnostic complexity of DADA2. Our patient exhibited several hallmark features, including persistent bi-cytopenia, recurrent febrile seizures, and eczematous skin rashes. Although eczematous rash is not a classical feature of DADA2, it may represent a nonspecific manifestation of immune dysregulation described in this condition. However, neonatal and infantile hematologic-predominant initial presentation of DADA2, as seen in our patient, remains underrecognized, which may lead to delays in diagnosis, particularly in the absence of classical vasculitis features. The identification of a novel mutation in the ADA2 gene (c.1442+2T>A variant) expands the known genetic variants associated with this disease and provides further insight into its pathogenesis.

The diagnosis of DADA2 is particularly challenging due to its rarity and the broad spectrum of clinical manifestations. In our patient, the early presentation with hydrops fetalis, respiratory distress, and persistent bi-cytopenia prompted an extensive evaluation for hematologic and infectious causes, all of which were inconclusive. The presence of hydrops fetalis in the neonatal period may represent an early and atypical manifestation of DADA2, although this association is not well described in the literature. It may reflect early severe systemic inflammation or hematologic dysfunction related to the disease process. Differential diagnoses considered prior to genetic confirmation included hematologic, infectious, and immune-mediated etiologies. Because ADA2 enzyme activity testing - which can support the diagnosis of DADA2 - is not available in our institution and given the concerning clinical picture with significant bi-cytopenia, early genetic testing with WES was pursued. This ultimately identified the causative ADA2 mutation and confirmed the diagnosis. The c.1442+2T>A variant is a splice-site variant with predicted disruption of canonical splicing. No RNA or protein functional studies were performed due to local unavailability. Its classification as likely pathogenic was based on the homozygous state, rarity, predicted functional impact, and phenotype consistent with DADA2. This case emphasizes the importance of considering genetic disorders such as DADA2 in patients with unexplained cytopenias and systemic inflammation, particularly when standard investigations fail to reveal an etiology. In our patient, alpha-thalassemia trait was considered an incidental finding and did not account for the severity of anemia, associated neutropenia, or overall clinical presentation.

Given the absence of randomized clinical trials for DADA2 management, treatment recommendations are based on case reports, case series, and expert opinions. TNF inhibitors, such as infliximab and etanercept, have shown efficacy in reducing systemic inflammation and preventing strokes, making them the cornerstone of DADA2 treatment [7]. As etanercept is generally not recommended for those below two years of age, our patient was initially started on infliximab infusions and received six doses at four-week intervals. Subsequently, due to family compliance challenges related to the long distance from our hospital, he was transitioned to subcutaneous etanercept to improve treatment adherence and convenience. Following treatment initiation, he demonstrated clinical stability with improvement in hematologic parameters and absence of any new systemic manifestations during follow-up. Ongoing monitoring continued in outpatient setting. TNF inhibitors remain the mainstay of therapy for patients with vasculitis or inflammatory phenotype, providing effective control of vascular and systemic manifestations. Additionally, allogenic HSCT has been used in severe cases with bone marrow failure or refractory vasculitis, showing significant improvements in clinical outcomes and providing curative hope for these patients [6,8]. Looking forward, emerging approaches such as gene therapy and enzyme replacement therapy are under investigation and may provide additional therapeutic options in the future.

The autosomal recessive inheritance pattern of DADA2 necessitates genetic counseling and family screening, as early identification of carriers and affected individuals can allow for timely interventions and prevention of serious consequences. In this case, the parents were extensively counseled about the rarity of the condition, its genetic inheritance, and the importance of screening all siblings and symptomatic family members. At the time of reporting, genetic screening of siblings had been planned and was pending.

This case is notable for its early neonatal onset with hydrops fetalis and a predominantly hematologic presentation in the absence of classical vasculitis features, which may delay diagnosis. In addition, the identification of a novel ADA2 mutation (c.1442+2T>A) expands the genetic spectrum of DADA2. To our knowledge, this is the first reported case from Oman and may reflect under-recognition of DADA2 in some populations.

The novel ADA2 mutation identified in our patient highlights the need for ongoing research into the genetic landscape of DADA2. Understanding the full spectrum of genetic mutations associated with the disease will support the advancement of more targeted and effective treatments. Future studies are needed to establish standardized diagnostic criteria and treatment protocols through collaborative international efforts and clinical trials.

## Conclusions

This case report underscores the importance of considering DADA2 in patients with unexplained cytopenias and systemic inflammation. The identification of a novel ADA2 mutation in this patient enriches the growing understanding of the genetic diversity and pathogenesis of this condition. A multidisciplinary approach, including genetic testing, comprehensive clinical evaluation, and targeted treatment, is essential for managing this rare disorder. Genetic counseling and family screening play a crucial role in early diagnosis and intervention, markedly improving patients’ outcome. This case highlights the need for continued research and international collaboration to enhance our understanding and management of DADA2.
